# Superficial Femoral Artery Spontaneous Pseudoaneurysm Revealing Pleomorphic Vascular Wall Sarcoma

**DOI:** 10.1002/ccr3.72310

**Published:** 2026-04-24

**Authors:** Kamal Majed, Kaleem Mohammad Basharat, Hajar M. Anas Nassani, Suha Turkmen

**Affiliations:** ^1^ Emergency Department Hamad Medical Corporation Doha Qatar; ^2^ College of Medicine, Qatar University Doha Qatar

**Keywords:** femoral artery, leiomyosarcoma, neoplasms, pulmonary embolism, vascular tissue

## Abstract

Vascular malignancy may rarely present as soft tissue swelling related to a spontaneous pseudoaneurysm. Our case report highlights the importance of prompt clinical examination, imaging, and surgical exploration for diagnosing vascular sarcoma presenting as a spontaneous pseudoaneurysm in a healthy patient.

## Graphical abstract

Spontaneous femoral artery pseudoaneursym with contrast media extrvasation.

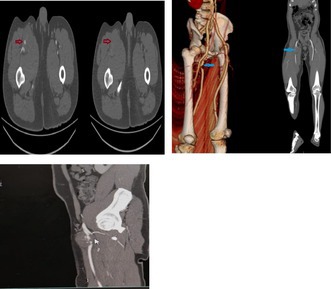

## 1 Introduction

A pseudoaneurysm, also referred to as a false aneurysm, is characterized by a contained rupture of the arterial wall resulting in a blood‐filled cavity located between the tunica media and tunica adventitia. This cavity maintains direct communication with the arterial lumen and is contained by the remaining arterial wall layers or adjacent soft tissues. Pseudoaneurysms most commonly arise secondary to iatrogenic injury or trauma and are frequently observed in the femoral artery, reflecting the high prevalence of vascular access procedures at this site, including catheterization and arterial puncture.

Spontaneous pseudoaneurysms occurring in the absence of trauma or iatrogenic injury are exceedingly rare and are typically associated with underlying arterial wall pathology. Reported etiologies include atherosclerosis, vasculitis, connective tissue disorders, and, less commonly, malignancy. Among malignant causes, vascular involvement by sarcomas, particularly leiomyosarcoma, represents an exceptionally rare etiology, as tumor infiltration may weaken the arterial wall, predisposing it to rupture and subsequent pseudoaneurysm formation.

In this report, we describe the case of a young adult male who presented with a spontaneous pseudoaneurysm of the superficial femoral artery, which was later found to be secondary to an infiltrating high‐grade leiomyosarcoma. His clinical course was further complicated by an acute pulmonary embolism, presenting a complex therapeutic challenge. This case highlights the need for a high index of suspicion, timely imaging, and a multidisciplinary approach to diagnosis and management.

### 1.1 Patient Presentation

A 41‐year‐old previously healthy male presented to the emergency department with a four‐day history of progressive swelling and pain in the right upper thigh. He denied any history of recent trauma, invasive vascular procedures, or systemic symptoms such as fever, weight loss, or night sweats. There was no personal or family history of vascular disease or malignancy.

On physical examination, the patient was hemodynamically stable. A firm, pulsatile mass approximately 4 cm in diameter was palpable in the anterior compartment of the right upper thigh. The overlying skin was intact, with no signs of erythema, warmth, or ulceration. Distal pulses were intact, and capillary refill was normal in the affected limb. No bruit was heard over the mass.

## 2 Differential Diagnosis, Investigations and Treatment

**FIGURE 1:**
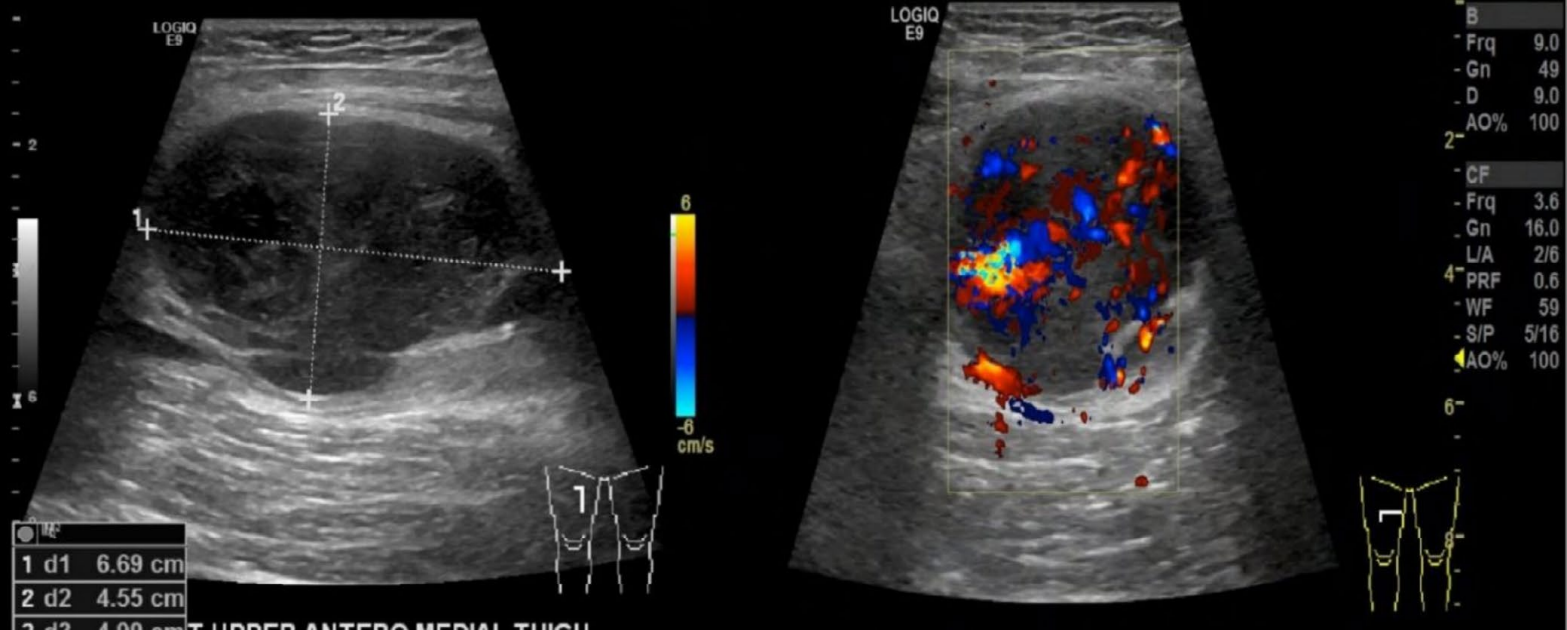
Ultrasound soft tissue: Well‐defined, hypoechoic, encapsulated lesion with internal vascularity, measuring 6.6 × 4.5 × 4.9 cm, consistent with a pseudoaneurysm.

**FIGURE 2:**
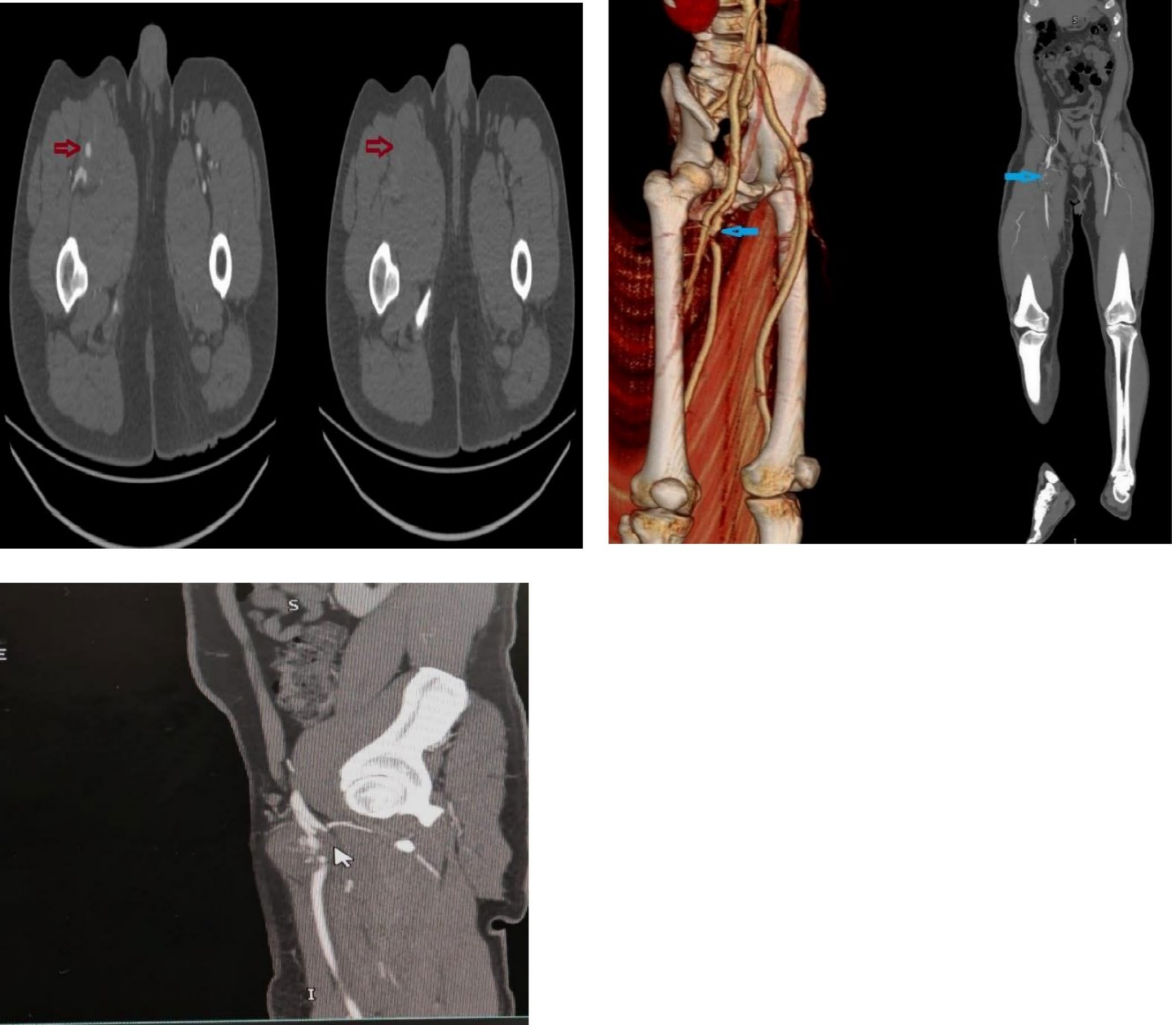
Computed tomography of lower limb: A 46 mm focal segment of the superficial femoral artery about 36 mm from its origin shows significant irregular luminal narrowing and mild posterior displacement. A 67 × 45 mm hematoma is noted around this segment predominantly along the anterior aspect with contrast ooze noted within.

Initial bedside ultrasound showed a well‐defined, hypoechoic, encapsulated lesion with internal vascularity, measuring 6.6 × 4.5 × 4.9 cm, consistent with a pseudoaneurysm (Figure 1). Computed tomography angiography of the lower limbs was performed and revealed a pseudoaneurysm of the superficial femoral artery with contrast extravasation (Figure 2). The absence of direct vascular injury and systemic symptoms raised the concern of vascular wall disease, especially malignancy. The patient was admitted under the vascular surgery team for further evaluation and surgical planning.

Within 24 h of admission, the patient developed sudden dyspnea, palpitations, and syncope. Blood pressure dropped to 97/65 mmHg with tachycardia around 120 bpm. He responded well to fluid resuscitation and oxygen therapy. Due to acute clinical deterioration, a CT pulmonary angiogram (CTPA) was urgently performed, revealing extensive bilateral pulmonary emboli with signs of right ventricular strain, including interventricular septal bowing and right heart dilation. He was transferred to the intensive care unit (ICU), and therapeutic anticoagulation was started with low molecular weight heparin.

Over the following days, the patient's cardiopulmonary status stabilized. On hospital day 10, once deemed stable for surgery, the patient underwent elective vascular repair of the pseudoaneurysm. Intraoperatively, a friable, vascular mass was identified arising from and infiltrating the wall of the superficial femoral artery. The mass extended intraluminally and was associated with aneurysmal dilation. The lesion was excised en bloc, and vascular continuity was restored using a reversed saphenous vein interposition graft. An enlarged satellite lymph node was also excised.

Histopathological analysis revealed a high‐grade pleomorphic sarcoma consistent with leiomyosarcoma, exhibiting significant mitotic activity and vascular wall invasion. The lymph node showed reactive hyperplasia with no evidence of metastasis.

## 3 Conclusion and Results

The patient had an uneventful recovery and was referred to a multidisciplinary oncology team. Adjuvant radiotherapy was planned due to the aggressive histology and vascular involvement.

## 4 Discussion

This case represents a rare but critical vascular complication of soft tissue sarcoma: spontaneous pseudoaneurysm formation due to direct arterial wall infiltration. Leiomyosarcomas are malignant tumors originating from smooth muscle cells. Although they most commonly arise in the uterus, retroperitoneum, and extremities, vascular leiomyosarcomas—especially those involving large arteries—are exceedingly uncommon.

Spontaneous pseudoaneurysms are rare and often misdiagnosed due to their nonspecific presentation [1, 2, 3, 4]. Most pseudoaneurysms result from vascular injury following catheterization, trauma, or infection [5]. However, in the absence of such a history, a spontaneous pulsatile mass should raise concern for neoplastic infiltration or vasculitis. In this case, the patient's young age, absence of trauma, and ultrasound findings prompted further imaging and surgical exploration.

The concurrent presence of pulmonary embolism raised concern for an underlying systemic vascular disorder, causing arterial wall weakening and pseudoaneurysm formation, with associated inflammatory hypercoagulability predisposing to thromboembolic events. Although the absence of systemic manifestations argues against these diagnoses. Traumatic and iatrogenic causes, as well as locally infiltrative soft tissue tumors, were excluded based on clinical history and imaging findings. Primary vascular or adjacent soft tissue malignancies were also considered, as these may present with an indolent course and minimal systemic symptoms.

Pseudoaneurysms pose significant risks, including rupture, distal embolization, thrombosis, and local compression. In this case, the clinical course was complicated by the onset of pulmonary embolism, probably due to local compression of the femoral vein and the malignant source of the pseudoaneurysm. This sequence illustrates how vascular sarcomas can cause both local and systemic thromboembolic complications. The co‐occurrence of pulmonary embolism complicated the management strategy, as anticoagulation increases the bleeding risk, especially in vascular lesions. However, in this patient, the risk of not treating pulmonary embolism was considered higher than the bleeding risk, and careful monitoring allowed for safe perioperative management [6, 7].

CT angiography is the preferred imaging modality for evaluating suspected pseudoaneurysms and assessing arterial anatomy. However, CT alone may not distinguish between a pseudoaneurysm caused by trauma and one resulting from neoplastic infiltration [8]. MRI provides better soft tissue characterization and may offer clues regarding tumor presence and extension, but it is often underutilized due to limited availability or urgency of presentation [9].

Surgical repair is the preferred therapeutic approach for spontaneous pseudoaneurysms, particularly when the underlying etiology is unclear, as it allows definitive vascular repair while providing tissue for histopathological diagnosis.

Surgical resection remains the primary treatment for vascular tumors. In our case, a reversed saphenous vein graft was selected for arterial reconstruction because of its favorable long‐term patency and lower infection risk compared to synthetic grafts [10]. Andrea et al. [11] reported successful vascular reconstruction using venous or prosthetic grafts, achieving arterial patency in 100% of cases and venous patency in 80%. A multidisciplinary approach remains the cornerstone of successful revascularization, ensuring optimal outcomes with minimal complications. Adjuvant radiotherapy is typically recommended for high‐grade sarcomas, especially when large vessels are involved, to reduce the risk of local recurrence [12].

Leiomyosarcomas involving blood vessels are associated with a poor prognosis. Their five‐year survival rate ranges from 30% to 60%, depending heavily on factors such as tumor size, histological grade, presence of metastasis, and completeness of surgical resection [12]. In our case, prompt diagnosis and surgical resection, followed by adjuvant therapy, offered the potential for improved outcomes despite the aggressive nature of the disease.

## 5 Conclusion

This case highlights a rare but important differential diagnosis of a spontaneous pseudoaneurysm—arterial wall invasion by soft tissue sarcoma. Clinicians should maintain a high index of suspicion in young patients presenting with pulsatile soft tissue masses, particularly in the absence of trauma or intervention. Early imaging with Doppler ultrasound and CTA is crucial for diagnosis, while MRI may assist in assessing tumor extension. Furthermore, this case underscores the need for multidisciplinary collaboration in managing complex vascular and oncologic cases. The concurrent presence of pulmonary embolism and active bleeding posed a significant therapeutic challenge. Ultimately, early stabilization, timely surgery, and histopathological confirmation enabled appropriate oncologic management.

## Author Contributions

**Kamal Majed:** writing – original draft, writing – review and editing. **Kaleem Mohammad Basharat:** writing – review and editing. **Hajar M. Anas Nassani:** visualization. **Suha Turkmen:** supervision, validation, writing – review and editing.

## Funding

The publication of this case report is funded by Qatar National Library.

## Consent

Written informed consent was obtained from the patient(s) to publish this report in accordance with the journal's patient consent policy.

## Conflicts of Interest

The authors declare no conflicts of interest.

## Data Availability

The data that supports the findings of this study are available on request from the corresponding author.
